# Resting-state electroencephalogram in drug-free subjects with at-risk mental states who later developed psychosis: a low-resolution electromagnetic tomography analysis

**DOI:** 10.3389/fnhum.2024.1449820

**Published:** 2024-08-27

**Authors:** Yuko Higuchi, Shizuka Odagiri, Takahiro Tateno, Michio Suzuki, Tsutomu Takahashi

**Affiliations:** ^1^Department of Neuropsychiatry, Graduate School of Medicine and Pharmaceutical Sciences, University of Toyama, Toyama, Japan; ^2^Research Center for Idling Brain Science, University of Toyama, Toyama, Japan; ^3^Center for Clinical Training, Toyama University Hospital, Toyama, Japan; ^4^Uozu Shinkei Sanatorium, Toyama, Japan; ^5^Itoigawa Clinic, Niigata, Japan; ^6^Ariwawabashi Hospital, Toyama, Japan

**Keywords:** schizophrenia, at-risk mental state, clinical high risk, resting state electroencephalogram, power spectrum, biomarker, low-resolution brain electromagnetic tomography, positive and negative syndrome scale

## Abstract

**Background and objectives:**

Several studies have reported on the resting-state electroencephalogram (EEG) power in patients with schizophrenia, with a decrease in α (especially α2) and an increase in δ and β1 power compared with healthy control; however, reports on at-risk mental states (ARMS) are few. In this study, we measured the resting-state EEG power in ARMS, and investigated its features and the relationship between the power of the frequency bands and their diagnostic outcomes.

**Methods:**

Patients with ARMS who were not on any psychotropic medication and met the Comprehensive Assessment of At-Risk Mental State criteria were included. Patients who developed psychotic disorders were labeled as the ARMS-P group, while patients with ARMS who were followed up prospectively for more than 2 years and did not develop psychotic disorders were classified as the ARMS-NP group. EEGs were measured in the resting state, and frequencies were analyzed using standardized low-resolution brain electromagnetic tomography (sLORETA). Seven bands (δ, θ, α1, α2, β1–3) underwent analysis. The sLORETA values (current source density [CSD]) were compared between the ARMS-P and ARMS-NP groups. Clinical symptoms were assessed at the time of EEG measurements using the Positive and Negative Syndrome Scale (PANSS).

**Results:**

Of the 39 patients included (25 males, 14 females, 18.8 ± 4.5 years old), eight developed psychotic disorders (ARMS-P). The ARMS-P group exhibited significantly higher CSD in the β1 power within areas of the left middle frontal gyrus (MFG) compared with the ARMS-NP group (best match: X = −35, Y = 25, Z = 50 [MNI coordinates], Area 8, CSD = 2.33, p < 0.05). There was a significant positive correlation between the β1/α ratio of the CSD at left MFG and the Somatic concern score measured by the PANSS.

**Discussion:**

Increased β1 power was observed in the resting EEG before the onset of psychosis and correlated with a symptom. This suggests that resting EEG power may be a useful marker for predicting future conversion to psychosis and clinical symptoms in patients with ARMS.

## 1 Introduction

A high-risk psychosis strategy aims to improve early detection of psychosis and provide earlier and more efficient treatment (McGorry et al., 2008; Riecher-Rossler et al., 2006). An at-risk mental state (ARMS) for psychosis defined using semi-structured clinical interviews includes attenuated psychotic symptoms, brief limited psychotic episodes, and genetic risk and social decline (Yung et al., 1998). However, not all patients identified with ARMS using these criteria develop psychosis. A recent meta-analysis reported a transition-to-psychosis rate of only 23% (Salazar de Pablo et al., 2021). Thus, the current criteria result in several false positives. Differentiating between patients with ARMS who will transition to psychosis (ARMS-P) and those who will not develop psychosis (ARMS-NP) is crucial to avoid imposing unnecessary burdens on patients.

Electroencephalography (EEG) is a noninvasive, convenient and economical electrophysiological measurement technique that explores neuronal activity by placing electrodes on the scalp (Schoenberg, 2020). EEG has a high temporal resolution on the same millisecond scale as brain neural activity, enabling accurate acquisition of neural activity and characterization of functional changes during dynamic cerebral activity (Schoenberg, 2020). EEGs are available in large healthcare facilities and are widely used to reveal the etiology of various neuropsychiatric disorders.

Patients with ARMS-P are more severely impaired in certain neurological domains compared to patients with ARMS-NP; therefore, biomarker studies have been performed to identify patients with ARMS-P using several measurement tools. As for EEG, evoked potentials containing event-related potentials, particularly mismatch negativity, have been reported to be able to detect patients with ARMS-P (Bodatsch et al., 2015; Erickson et al., 2016). Traditional resting EEG findings have also been reported because they are easy to measure and important for understanding the functional characteristics of the idling human brain. The EEG raw signal can be decomposed into five oscillatory rhythms or broad frequency bands, namely δ (0.5–4.0 Hz), θ (4–8 Hz), α (8–13 Hz), β (13–30 Hz), and γ (30–100 Hz) bands (Babiloni et al., 2020). Previous studies for the power spectrum in schizophrenia generally reported increased δ (Guntekin and Basar, 2016; Newson and Thiagarajan, 2018) and decreased α (Newson and Thiagarajan, 2018; Murphy and Ongur, 2019; Begre et al., 2003; Pascual-Marqui et al., 1999), while inconsistent findings have been also reported in studies investigating abnormalities of the resting θ, β and γ band power (Newson and Thiagarajan, 2018; Perrottelli et al., 2021; Reilly et al., 2018). Some studies subdivided frequency bands into lower and higher frequency components, namely α1, β2 (Newson and Thiagarajan, 2018). Narrower frequency bands can reduce the risk of offsetting or undetected frequency effects (Klimesch et al., 1997; Li et al., 2020). By this approach, the resting-state EEG in patients with schizophrenia was reported with a decrease in α (especially α2) and increase in β1 power (Vignapiano et al., 2019). As for ARMS, within the authors’ knowledge, four papers have examined whether EEG power predicts future transition to psychosis. Zimmermann et al. (2010) showed difference between ARMS-P and ARMS-NP by combination with negative symptom, the δ, θ, β1, and β2 power, however, they failed that negative symptom or EEG spectral power alone predicted transition to psychosis (Zimmermann et al., 2010). Sollychin et al. (2019) tried to reveal increased frontal slow wave activity and functioning shows any value in the prediction of transition to psychosis, however, there was no difference between the ARMS-P and the ARMS-NP groups, and no change in slow frequency power following transition to psychosis (Sollychin et al., 2019). Ramyead et al. (2016) suggested that current source density (CSD, which will be mentioned later) measurements extracted from clinical resting state EEG can help to improve the prediction of psychosis on a single-subject level by using machine-learning (Ramyead et al., 2016). They also showed ARMS-P patients showed higher γ activity in the medial prefrontal cortex compared to healthy control and ARMS-P patients lagged phase synchronization of β oscillations decreased compared to ARMS-NP and healthy control (Ramyead et al., 2015). Although their suggestions were evocative, the results were varied and further evidence demanded. Thus, it remains elusive whether ARMS patients have abnormal resting-state EEG power and whether, if present, it is associated with their clinical characteristics (e.g., symptom severity, later psychosis onset).

Low-resolution electromagnetic tomography (LORETA) provides three-dimensional images of brain electrical activity, which is the so-called CSD (Pascual-Marqui et al., 1999). The strength of LORETA includes the better representation of a widely distributed or multiple-oriented activity, which is difficult to model with a dipole procedure. It is a simple method and produces less demand on patients than other functional brain measures, such as functional magnetic resonance imaging. EEG recording CSD measures have remarkable advantages over scalp potentials in terms of topographic patterns, strength of activation, and more reliable spatial localization of neuroelectric activity during resting EEG, as well as during cognitive tasks, by elucidating subtle differences across and within several neuropsychiatric conditions, such as schizophrenia (Kamarajan et al., 2015).

When investigating resting EEG, we must consider the weakness of its sensitivity to the effects of psychotropic drugs, such as benzodiazepines and antipsychotics (Mucci et al., 2006). It is important to conduct resting EEG studies in drug-free patients to identify reliable biomarkers.

In this study, we measured the resting-state EEG power using LORETA in psychotropic medication-free patients with ARMS, and investigated its features and the relationship between the power of the frequency bands and their outcomes. The relationship between resting EEG signals, clinical symptoms, and cognitive and social functioning in patients with ARMS was also investigated. We predicted that the frequency bands characteristic reported in schizophrenia would be found in patients with ARMS-P but not in patients with ARMS-NP.

## 2 Materials and methods

### 2.1 Participants

Patients with ARMS were recruited from the University of Toyama Hospital or the Toyama Prefectural Mental Health Centre (Mizuno et al., 2009). None of the patients took psychotropic medications, including anti-psychotics, anti-depressant, or benzodiazepines within 2 weeks before the EEG recording. Diagnoses were made by experienced psychiatrists based on the Comprehensive Assessment of At-Risk Mental State (Yung et al., 1996). Psychiatric and treatment histories were collected from the participants, and their families and medical records. Physical examination and standard laboratory tests confirmed that the eligible patients were physically healthy. Exclusion criteria included the following: patients with a history of substance abuse or dependence, seizure, and head injury.

Individuals with ARMS were further subgrouped based on clinical outcomes during the follow-up period as described in previous reports (Fusar-Poli et al., 2012). Conversion to psychosis was defined according to the psychotic disorder criteria in the Comprehensive Assessment of At-Risk Mental State: (i) hallucinations, unusual thoughts, and suspiciousness exceed defined severities, or delusion with strong conviction, or conceptual disorganization exceeds moderate level, (ii) frequency of symptoms is at least several times a week, and (iii) the episode is longer than 1 week (Yung et al., 2005).

Experienced psychiatrists administered the Positive and Negative Syndrome Scale (PANSS) (Kay et al., 1987). The Japanese Adult Reading Test (JART) (Matsuoka et al., 2006) was used to estimate premorbid IQ. The Brief Assessment of Cognition in Schizophrenia (BACS) (Keefe et al., 2004; Kaneda et al., 2007), Schizophrenia Cognition Rating Scale (SCoRS) (Keefe et al., 2006; Kaneda et al., 2011; Higuchi et al., 2017), and modified Global Assessment of Functioning (mGAF) (Hall, 1995) were used to evaluate cognitive and social functions. The BACS score was standardized using z-scores based on the mean scores of healthy Japanese controls (Kaneda et al., 2007).

This study was conducted in accordance with the principles of the Declaration of Helsinki and approved by the Committee on Medical Ethics of Toyama University (no. I2013006) on February 5, 2014. Written informed consent was obtained from all participants after a full explanation of the purpose and procedure of the study was provided. Written consent was obtained from the parents or guardians of participants under 20 years of age.

### 2.2 EEG recording

EEGs were recorded at baseline, based on previous reports from our laboratory (Higuchi et al., 2008; Sumiyoshi et al., 2009; Miyanishi et al., 2013). Briefly, a 32-channel DC amplifier (EEG-2100 version 2.22 J, Nihon Kohden Corp., Tokyo, Japan) was used according to the international 10–20 system. Recordings were performed using electro cap (Electro cap Inc., Eaton, OH) in a sound-attenuated room. The data were collected at a sampling rate of 500 Hz. All electrodes were referred to as the average amplitude of the ear electrodes (bandwidth, 0.53–120 Hz, 60 Hz notch filter). Electrode impedance was < 10 k-ohm. Patients were assessed using the Epworth Sleepiness Scale (Johns, 1991) and were required to lay on a bed for 5 min with their eyes closed in a silent and dark room during EEG recording. From the resting-EEG, 2-second, 20-epoch EEGs without large artifacts, such as blinking, were extracted manually.

### 2.3 Standardized LORETA (sLORETA) analysis

The sLORETA images were obtained by estimating the CSD distribution for epochs of brain electrical activity on a dense grid of 6239 voxels at 5-mm spatial resolution applied to the digitized Talairach and Tournoux (1988), based on the established method (Pascual-Marqui et al., 1999). The sLORETA makes use of the three-shell spherical head model registered in the Talairach atlas, which is available as a digitized magnetic resonance imaging from the Brain Imaging Centre, Montreal Neurologic Institute. The registration between spherical and realistic head geometries uses EEG electrode coordinates (Towle et al., 1993). The solution space was restricted to the cortical gray matter and hippocampus, as determined by the corresponding digitized Probability Atlas, which is also available from the Brain Imaging Center.

Cross-spectra of the EEG epochs for each subject in each condition were computed using the sLORETA software in eight frequency bands: δ, 1.5–6.0 Hz; θ, 6.5–8.0 Hz; α1, 8.5–10.0 Hz; α2, 10.5–12.0 Hz; β1, 12.5–18.0 Hz; β2, 18.5–21.0 Hz; and β3, 21.5–30.0 Hz. The sLORETA computed the cortical distribution of the CSD of neuronal oscillations in 6239 voxels from the averaged cross-spectra in each band for each subject under each condition. The CSD data were normalized subject-wise; the CSD at each voxel was normalized with the power density averaged across all frequencies and all 6239 voxels (Babiloni et al., 2007). Subsequently, subtracted CSD were compared between the ARMS-P and -NP groups voxel-by-voxel in each band using the t-statistical non-parametric mapping implemented in sLORETA, and t-values were obtained.

Significant voxels were counted in each brain region as defined by the sLORETA. The results showed that all voxels with significant differences were contained in the left middle frontal gyrus (MFG). With reference to this, we calculated the average CSD containing the following brain regions of interest: significant 3-voxels ([−35, 25, 50], [−35, 25, 45], and [−35, 20, 50]), left MFG, left Brodmann area 8, and left frontal lobe. In the correlation analysis, we used these CSDs divided by the average CSD of the occipital α band, which is the basic rhythm.

### 2.4 Data analysis

Statistical analyses were performed using the Statistical Package for Social Sciences version 25 (SPSS Japan Inc.) and Jamovi Software version 2.3.18.0.^1^ Demographic and clinical data (age, JART, PANSS, BACS, SCoRS, and mGAF scores) were assessed using independent t-tests. Age had skewed distributions, and the nonparametric Mann-Whitney U (for two-group comparisons) test was used to compare group differences. Sex was analyzed using the Chi-square test. Comparisons between the ARMS-P and -NP groups in LORETA source imaging were conducted using voxel-by-voxel unpaired t-statistics after logarithmic transformation of the data. The Holmes’ non-parametric correction was applied for multiple comparisons (Holmes et al., 1996). The relationships between CSD and PANSS, BACS, SCoRS, and mGAF were analyzed using Spearman’s rank correlations because CSD had skewed distributions. The Benjamini–Hochberg false discovery rate procedure was used for post-hoc analysis (Benjamini et al., 2001). Significance was set at a value of p-value of less than 0.05 (two-tailed) for CSD analysis and false discovery rate adjusted p-value (= *q*) for correlation analysis.

## 3 Results

### 3.1 Patient demographics

The demographic and clinical data of the participants at baseline are shown in Table 1. Briefly, this study included participants with an average age of 18.8 ± 4.5 years old, 64% male and estimated premorbid IQs measured by the JART were over 70. During the follow-up period of the 39 ARMS patients, eight (20.5%) developed psychosis (ARMS-P) with the definitive diagnoses of schizophrenia (n = 7) and depression with psychotic symptoms (n = 1). Thirty-one patients did not develop psychosis during the 2-year follow-up period and were classified as ARMS-NP. Age, sex ratios, estimated premorbid IQ measured by the JART, and PANSS scores did not differ significantly between the ARMS-P and -NP groups. Regarding cognitive function, the ARMS-P indicated over one standard deviation lower on a BACS composite score. The impairment of BACS in the ARMS-NP group was milder, and the difference from the ARMS-P group was at the trend level (p = 0.06). The SCoRS score was high in the current dataset, particularly for the ARMS-P group, and there was a significant difference between the ARMS-P and -NP groups (p = 0.006). Social functioning, measured using the mGAF, was approximately 40 points, and no significant differences were found between the groups.

**TABLE 1:** Patient demographics and clinical data.

	ARMS-NP	ARMS-P	Group difference^a^
	*n* = 31	*n* = 8	
Age (years)	18.5 (3.8)	20.2 (6.9)	U_31,8_ = 123, *p* = 0.98
Gender (male/famale)	20/11	5/3	χ^2^ = 0.01, *p* = 0.91
JART	96.4 (10.9)	93.0 (11.0)	t_28,8_ = 0.76, *p* = 0.45
PANSS: positive	13.4 (3.9)	13.9 (2.2)	t_30,8_ = −0.36, *p* = 0.72
PANSS: negative	18.8 (7.1)	19.3 (4.4)	t_30,8_ = −0.18, *p* = 0.86
PANSS: general psychopathology	34.3 (9.3)	35.6 (8.0)	t_30,8_ = −0.38, *p* = 0.71
:total	66.4 (16.8)	68.8 (12.7)	t_30,8_ = −0.37, *p* = 0.72
BACS^b^	−0.7 (1.0)	−1.5 (1.2)	t_31,8_ = 1.92, *p* = 0.06
SCoRS^c^	4.3 (2.0)	6.6 (2.1)	t_29,8_ = −2.92, ***p* = 0.006**
mGAF^d^	49.4 (8.4)	42.4 (9.0)	t_22,7_ = 1.89, *p* = 0.07

All values are shown as means (standard deviations). Bold letter indicates that there was a significant difference (*p* < 0.05).

^a^The demographic differences between the groups were examined using the Mann–Whitney U test, or Student’s t-test, or chi-square (χ2) test.

^b^The BACS composite score was calculated by averaging all the z-scores of the six primary measures from the BACS.

^c^The data ranged from 0 to 10, with larger numbers representing worse function.

^d^The data ranged from 0 to 100, wherein healthy participants had scores ranging from 90 to 100 points. ARMS-NP, at-risk mental state non-psychosis; ARMS-P, at-risk mental state psychosis; BACS, Brief Assessment of Cognition in Schizophrenia; JART, Japanese Adult Reading Test; mGAF, modified Global Assessment Functioning; PANSS, Positive and Negative Syndrome Scale; SCoRS, Schizophrenia Cognition Rating Scale.

### 3.2 Brain CSD activity

**FIGURE 1:**
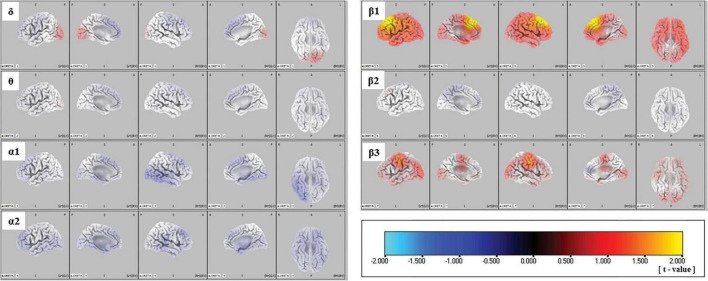
Comparison of source imaging in the ARMS-P and -NP groups, as revealed by SnPM voxel-wise sLORETA comparisons of independent samples. Negative (i.e., lower in ARMS-P group) to positive (i.e., higher in ARMS-P group) t-values are represented by cyan-blue-black-red-yellow. ARMS-NP, at-risk mental state non-psychosis; ARMS-P, at-risk mental state psychosis; sLORETA, standardized low-resolution brain electromagnetic tomography; SnPM, statistical non-parametric mapping.

**FIGURE 2:**
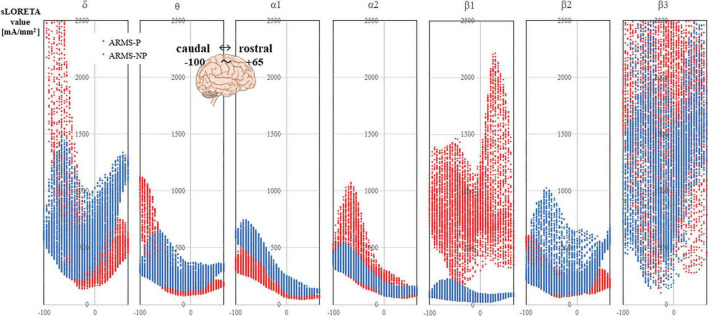
The CSDs of the ARMS-P (red marker) and ARMS-NP (blue marker) groups are plotted. The X-axis of each graph represents the Y-axis of the MNI coordinate, which ranged from –100 to +65. The negative direction is caudal and the positive direction is rostral. ARMS-NP, at-risk mental state non-psychosis; ARMS-P, at-risk mental state psychosis; CSD, current source density; MNI, Montreal Neurological Institute.

The sLORETA images for each frequency band are shown in Figure 1. The average CSDs of the ARMS-P and -NP groups for each voxel are plotted in Figure 2. The mean CSDs of the ARMS-P group seem larger in the α2 (especially in the caudal area) and β1 (rostral area) bands. On the other hand, the CSDs of the α1 band in ARMS-P looks smaller especially in the caudal area. Table 2 shows the brain areas with the five largest differences in CSD for each frequency band. Statistical analysis revealed that the ARMS-P group had significantly higher CSDs for β1 at 3 regions within the left MFG compared to the ARMS-NP group. In Supplementary Figure 1, the t-values of each voxel calculated using the sLORETA software and significant voxels (indicated by arrows) are plotted.

**TABLE 2:** MNI coordinates for brain areas showing the largest CSD differences (top five) between the ARMS-P and -NP groups.

	Rank	*t*-value	MNI coordinate			Brodmann area	Brain Structure	*p*
			X	Y	Z			
δ	1	0.8620	−15	−90	10	18	Left Middle Occipital Gyrus	>0.1
	2	0.8610	−15	−90	15	18	Left Middle Occipital Gyrus	>0.1
	3	0.8600	−20	−90	15	18	Left Cuneus	>0.1
	4	0.8580	−15	−85	10	18	Left Cuneus	>0.1
	5	0.8560	−20	−95	15	18	Left Cuneus	>0.1
θ	1	0.2675	−25	−95	15	18	Left Middle Occipital Gyrus	>0.1
	2	0.2647	−25	−95	20	19	Left Cuneus	>0.1
	3	0.2640	−30	−95	15	19	Left Middle Occipital Gyrus	>0.1
	4	0.2629	−20	−90	15	18	Left Cuneus	>0.1
	5	0.2622	−25	−95	10	18	Left Middle Occipital Gyrus	>0.1
α1	1	0.1430	−35	−50	65	5	Left Postcentral Gyrus	>0.1
	2	0.1410	−40	−50	60	40	Left Inferior Parietal Lobule	>0.1
	3	0.1410	−35	−50	60	5	Left Postcentral Gyrus	>0.1
	4	0.1400	−35	−55	60	40	Left Inferior Parietal Lobule	>0.1
	5	0.1360	−30	−60	60	7	Left Superior Parietal Lobule	>0.1
α2	1	0.0436	25	−55	70	7	Right Postcentral Gyrus	>0.1
	2	0.0384	20	−55	70	7	Right Postcentral Gyrus	>0.1
	3	0.0344	25	−65	65	7	Right Superior Parietal Lobule	>0.1
	4	0.0316	25	−60	65	7	Right Superior Parietal Lobule	>0.1
	5	0.0308	15	−55	70	7	Right Postcentral Gyrus	>0.1
β1	**1**	**2.3300**	−**35**	**25**	**50**	**8**	**Left Middle Frontal Gyrus**	**<0.05**
	**2**	**2.3200**	−**35**	**25**	**45**	**8**	**Left Middle Frontal Gyrus**	**<0.05**
	**3**	**2.3100**	−**35**	**20**	**50**	**8**	**Left Middle Frontal Gyrus**	**<0.05**
	4	2.3000	−40	25	45	8	Left Middle Frontal Gyrus	<0.1
	5	2.3000	−30	20	45	8	Left Middle Frontal Gyrus	<0.1
β2	1	0.3990	−40	20	50	8	Left Middle Frontal Gyrus	>0.1
	2	0.3890	−35	20	50	8	Left Middle Frontal Gyrus	>0.1
	3	0.3770	−35	15	50	8	Left Middle Frontal Gyrus	>0.1
	4	0.3730	−35	20	40	9	Left Precentral Gyrus	>0.1
	5	0.3670	−40	25	45	8	Left Middle Frontal Gyrus	>0.1
β3	1	1.5800	60	−10	45	6	Right Precentral Gyrus	>0.1
	2	1.5500	60	−15	40	4	Right Precentral Gyrus	>0.1
	3	1.5500	60	−15	45	6	Right Precentral Gyrus	>0.1
	4	1.5400	65	−15	35	4	Right Precentral Gyrus	>0.1
	5	1.5300	60	−10	40	6	Right Precentral Gyrus	>0.1

The *t*-values were calculated based on the CSD results of the ARMS-P and ARMS-NP groups. Bold letters indicate the voxels with significant group differences (*p* < 0.05). ARMS-NP, at-risk mental state non-psychosis; ARMS-P, at-risk mental state psychosis; CSD, current source density; MNI, Montreal Neurological Institute.

### 3.3 Relationships between psychiatric or cognitive symptoms and CSD activity

Table 3 shows the relationships between the β1/α ratio of the CSD at the left MFG and PANSS in patients with ARMS (n = 39). Statistical analyses by Spearman’s rank correlation revealed that the CSD significantly and positively correlated with the PANSS G1 score (General Psychopathology; G1 = Somatic concern). It was with moderate to large effect sizes (rho = 0.50, R^2^ = 0.25, p = 0.0017, q = 0.027). When analyzed separately for ARMS-P and ARMS-NP, only ARMS-NP correlated with G1 (rho = 0.53, R^2^ = 0.28, p = 0.0023, q = 0.037), and it was also a moderate to large effect size. We also investigated the correlations between the cognitive and social functioning of patients and the β1/α ratio of the CSD at the left MFG, but the results showed no significant correlations (Supplementary Table 1). In the other regions of interest, the β1/α CSD values did not relate to clinical data (data not shown).

**TABLE 3:** Relationships between the β/α ratio of the CSD at the left MFG and PANSS in patients with ARMS.

		rho		R^2^	*p*
PANSS total		0.28		0.08	0.09
PANSS positive		0.05		0.00	0.75
PANSS negative		0.27		0.07	0.11
PANSS general psychopathology		0.21		0.04	0.20
		**rho**	**R^2^**	***p***	***q***
PANSS positive	P1	0.08	0.01	0.63	4.40
	P2	0.08	0.01	0.63	1.47
	P3	−0.04	< 0.01	0.81	1.42
	P4	0.00	< 0.01	0.99	0.99
	P5	0.02	< 0.01	0.89	1.04
	P6	0.03	< 0.01	0.87	1.22
	P7	−0.08	0.01	0.63	2.21
PANSS negative	N1	0.40	0.16	0.01	0.10
	N2	0.12	0.01	0.49	1.14
	N3	0.07	< 0.01	0.70	0.97
	N4	0.08	0.01	0.66	1.15
	N5	0.03	< 0.01	0.88	1.03
	N6	0.40	0.16	0.02	0.05
	N7	−0.02	< 0.01	0.92	0.92
PANSS general psychopathology	**G1**	**0.50**	**0.25**	**0.0017**	**0.027**
	G2	0.23	0.05	0.18	0.93
	G3	−0.04	< 0.01	0.79	0.85
	G4	0.06	< 0.01	0.72	0.82
	G5	0.12	0.01	0.48	1.09
	G6	0.09	0.01	0.61	0.89
	G7	0.19	0.04	0.27	0.87
	G8	−0.02	< 0.01	0.90	0.90
	G9	−0.16	0.03	0.35	0.94
	G10	0.11	0.01	0.51	1.02
	G11	0.25	0.06	0.13	1.04
	G12	−0.08	0.01	0.65	0.80
	G13	−0.10	0.01	0.56	1.00
	G14	0.20	0.04	0.23	0.91
	G15	0.08	0.01	0.63	0.84
	G16	0.09	0.01	0.60	0.95

Values were compared using the Spearman’s rank correlation coefficient. Bold letter indicates that false discovery rate adjusted *p*-value (*q*) < 0.05. PANSS positive: P1, Delusions; P2, Conceptual disorganization, P3, Hallucinations; P4, Excitement; P5, Grandiosity; P6, Suspiciousness/persecution; and P7, Hostility. PANSS negative: N1, Blunted affect; N2, Emotional withdrawal; N3, Poor rapport; N4, Passive/apathetic social withdrawal; N5, Difficulty in abstract thinking; N6, Lack of spontaneity and flow of conversation; and N7, Stereotyped thinking. PANSS general psychopathology: G1, Somatic concern; G2, Anxiety; G3, Guilt feelings; G4, Tension; G5, Mannerisms and posturing; G6, Depression; G7, Motor retardation; G8, Uncooperativeness; G9, Unusual thought content; G10, Disorientation; G11, Poor attention; G12, Lack of judgment and insight; G13, Disturbance of volition; G14, Poor impulse control; G15, Preoccupation; G16, Active social avoidance ARMS, at-risk mental state; CSD, current source density; MFG, middle frontal gyrus; PANSS, Positive and Negative Syndrome Scale.

## 4 Discussion

Our current resting-EEG investigation revealed that the ARMS-P group had a significantly higher β1 power within areas of the left MFG compared with the ARMS-NP group. The advantage of our study was a simple and well understandable finding that β1 power alone predicts the transition to psychosis. This finding suggests that the features of the resting EEG that are impaired during psychosis exist prior to the onset of psychosis as a trait marker. The CSD of the left MFG was positively correlated with psychiatric symptoms, suggesting a relationship between the clinical phenotype and underlying neuropsychological mechanisms. These findings suggest that the resting EEG power may be a useful marker for predicting future diagnoses and clinical symptoms in patients with ARMS.

As for schizophrenia, abnormalities of β power manifest as increased activity in the resting-state EEG (Venables et al., 2009; Narayanan et al., 2014; Garakh et al., 2015). However, some studies found no differences in the resting β power between patients with schizophrenia and controls (Renaldi et al., 2019; Harris et al., 2006; Yeragani et al., 2006; Clementz et al., 1994). To the best of our knowledge, no study has reported that resting β power is reduced in schizophrenia. The β oscillation is thought to be associated with sensorimotor functions (Hari and Salmelin, 1997; Pfurtscheller and Lopes da Silva, 1999), the vigilant and excited state of the brain (Li et al., 2020; Pfurtscheller and Lopes da Silva, 1999; Al-Ezzi et al., 2020), and cognitive processes, such as the working memory and top-down regulation of attention (Brinkman et al., 2014; Spitzer and Haegens, 2017; Engel et al., 2001; Miskovic et al., 2010). Some studies found correlations between the β power and clinical symptoms, such as worse negative symptoms (Zimmermann et al., 2010; Gschwandtner et al., 2009) and decreased insight (Arikan et al., 2018). The findings of increased β power in the ARMS-P group in the present study are consistent with findings in previous studies of schizophrenia, suggesting that they share the same abnormalities in brain function, which were detected in schizophrenia, compared with the ARMS-NP group. In the current study, a somatic concern measured by PANSS positively correlated with the CSD of the β1-band. The somatic concern itself does not differ between ARMS-P and ARMS-NP (data not shown), and this finding might relate to biological background as a clinical high-risk condition for psychiatric disorders rather than a trait of psychosis. Somatic concerns refer to experiences of somatic symptoms that may be caused by an underlying somatic background, but can also occur in patients with anxiety disorder, depression, stress-related disorder, or psychosomatic symptoms (Tolmunen et al., 2014; Sikharulidze et al., 2017). The frontal lobe has functions such as motor function, thinking, decision-making, emotional control, memory retention, behavioral control, in particular the MFG is known to be involved in reorienting attention(Shulman et al., 2009; Japee et al., 2015). Also, the β band activity, particularly in lower β activity range (β1), is described to link to active cognitive engagement and vigilance, indicating heightened alertness and mental effort during problem-solving and decision-making tasks (Ray and Cole, 1985). ARMS subjects with high β1 power at MFG might have abnormal switching of attention to the somatic concern. Recently, a report indicated that patients with anxiety disorder exhibited a significant increase in the β rhythm and decrease in the α1 rhythm of the power spectral density (Shen et al., 2022). According to that study, although the α1 rhythms also had statistically significant differences in the power spectral density analysis, the diagnostic classification accuracy of the α1 rhythms (74%) was lower compared to the β rhythms (96%). That finding corresponded with the present study, which showed that β abnormality was more severe than α abnormality and a correlation existed between a high β/α ratio and severe anxiety-related symptoms. One possible explanation for this finding may be increased worry or internal cognitive processing of anxiety in the form of divergent, negatively biased mind wandering during nonspecific information processing (Schoenberg, 2020). Thus, significant changes in the rhythm might help explain the neural mechanism of ARMS and provide basic theoretical support for biomarker identification. Compared with previous studies discussing the differences between ARMS-P and ARMS-NP, our study partially agreed with the study by Zimmermann et al. (2010) who found differences in β1 power, within combination of other frequency bands (Zimmermann et al., 2010), and with the study of Sollychin et al. (2019) who found no difference in the slow wave activity (Sollychin et al., 2019). Ramyead et al. (2015), employed LORETA for quantitative EEG similar to us, also found differences in β oscillations for phase synchronization (Ramyead et al., 2015). Thus, the β-power might be a key frequency in predicting psychotic transitions in ARMS, as some studies found findings related to the β band as we showed. However, the consistent results were partial, and the four previous studies performed comparison in only a small number of ARMS-Ps (*n* = 7–23), suggesting that more detailed studies with more big data accumulation were needed.

In Figure 2, the mean CSDs of the ARMS-P group seemed larger in the α2 but smaller in the α1 compared with the ARMS-NP group. However, we failed to detect significant group difference related to α activity, probably due to the small sample size; hence, further investigation is needed. The α amplitude in the posterior electrodes increases in relaxed situations when the eyes are closed (Treder et al., 2011) and is thought to reflect an idling state of the brain (Bazanova and Vernon, 2014). This notion may be in line with increased β power, which reflects the vigilant and excited state of the brain (Li et al., 2020; Pfurtscheller and Lopes da Silva, 1999; Al-Ezzi et al., 2020), in patients with ARMS-P. It is well-known that the amplitude of the α frequency band is related to the synchrony of the underlying neuro-electrical source and a reduction in α wave amplitude is often labeled as desynchronization (Pfurtscheller and Lopes da Silva, 1999). Hence, the amplitude of the α rhythm is an index of cortical inactivity that is thought to be, in part, generated by the thalamus (Bazanova and Vernon, 2014). As for schizophrenia, many studies have shown that a reduction in α, particularly of α2, is thought to reflect reduced brain function (Galderisi et al., 1994; Sponheim et al., 1994; Boutros et al., 2008; Begic et al., 2011). A finding of negative correlation between α1 spectral amplitude and the disorganization factor measured by PANSS in schizophrenia (Vignapiano et al., 2019) may also support such mechanism.

While there is consensus on the EEG power spectrum findings in schizophrenia, there are also some contradictory findings. This is probably due to the heterogeneity of schizophrenia, which includes interindividual variability that has not yet been sufficiently resolved (Strik et al., 2017). Moreover, in contrast to this study, some research have revealed that resting EEG power did not differ between ARMS-P and -NP (Zimmermann et al., 2010; Lavoie et al., 2012; Ranlund et al., 2014). There are possible reasons why the findings in the ARMS-P group in this study were not consistent with these previous ARMS findings, such as high heterogeneity of ARMS-P individuals, rather mild EEG abnormalities in ARMS than in established schizophrenia (Bazanova and Vernon, 2014), and potential influence of antipsychotic and other medications (Treder et al., 2011). Nevertheless, a report by Zimmermann et al. (Brinkman et al., 2014) might partly support our findings in showing that resting-state EEG could be predictive of psychosis onset in combination with negative symptoms.

A major limitation of this study was the lack of healthy comparison subjects, where we used the ARMS-NP group as a control group for the ARMS-P group. Further, the number of participants was relatively small, which may have limited the generalizability and statistical power of the results. Finally, the ARMS-P group included a patient who was later definitively diagnosed with depression; this may increase the heterogeneity of our ARMS-P group.

In conclusion, the present study suggested the ability of β1 power to predict the development of psychosis in vulnerable subjects. Our observation also confirmed that the β/α ratio was associated with clinical symptoms related to physical concern. For a biomarker to be actionable, it must be clinically predictive at the individual level and viable in the clinical setting (Abi-Dargham et al., 2023). Therefore, it is important that findings that can be used as biomarkers were detected in the resting EEG, which can be easily measured.

## Data Availability

The raw data supporting the conclusions of this article will be made available by the authors, without undue reservation.

## Footnotes

1
https://www.jamovi.org
