# Supplementary material for: Resting-state electroencephalogram in drug-free subjects with at-risk mental states who later developed psychosis: a low-resolution electromagnetic tomography analysis
Source: Front Hum Neurosci. 2024 Aug 27;18:1449820. doi: 10.3389/fnhum.2024.1449820 (PMC11384587; doi:10.3389/fnhum.2024.1449820)
Supplement: Supplementary file 1 [file Data_Sheet_1.PDF]

Suppl 1

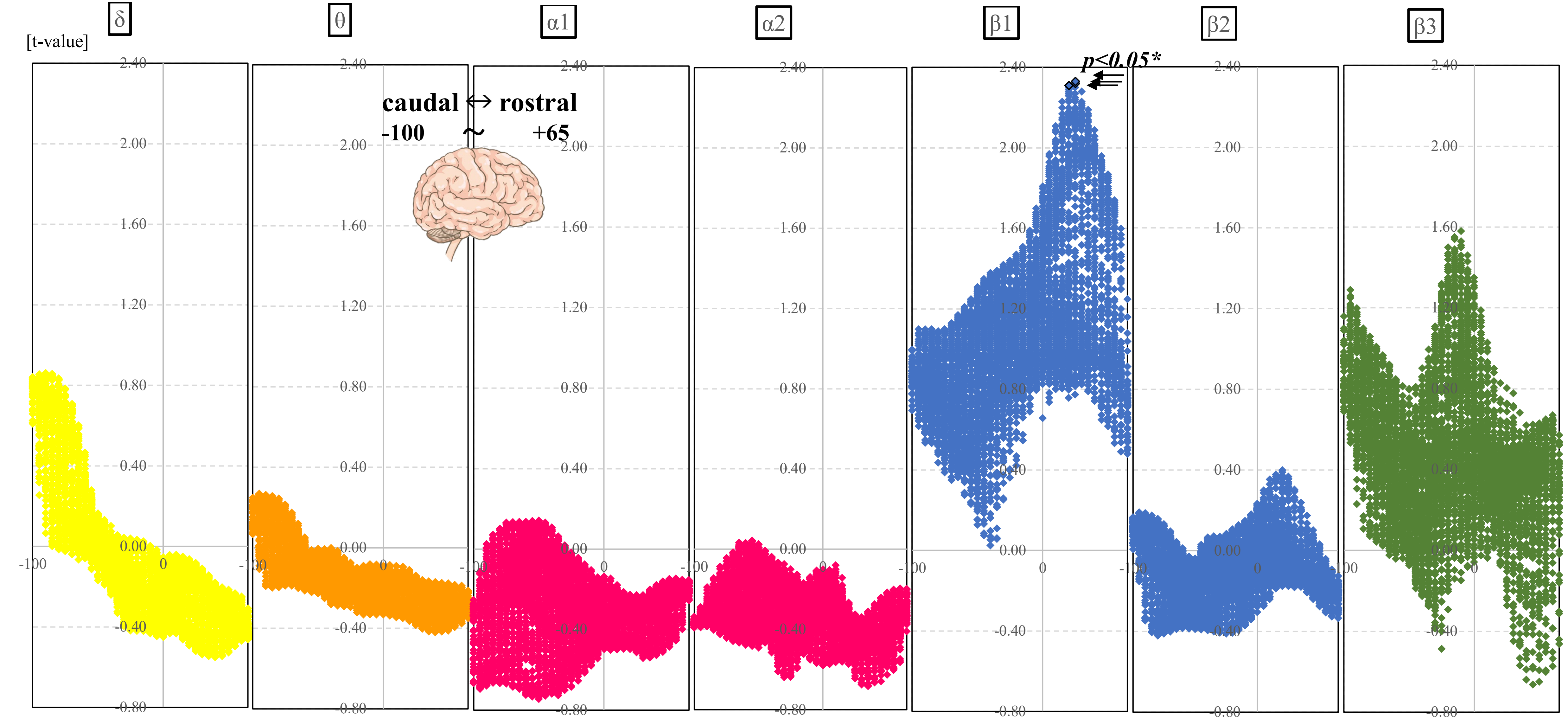

Note: The CSDs of ARMS-P and ARMS-NP were t-tested, and the t-values are plotted. The X-axis of each graph represents the Y-axis of the MNI coordinate, which ranged from -100 to +65. The negative direction is caudal and the positive direction is rostral.

Abbreviate: ARMS-NP, at-risk mental state non-psychosis; ARMS-P; at-risk mental state psychosis; CSD, current source density; MNI, Montreal Numerological Institutes.

Suppl 2

Relationships between the β/α ratio of CSD at left MFG and social/cognitive functions in ARMS patient

	rho	p
BACS composite score	0.08	0.63
SCoRS	0.01	0.96
mGAF	0.26	0.19

		rho	p
BACS subscore	verbal memory	0.10	0.56
	woeking memory	-0.01	0.94
	motor function	0.15	0.37
	verbal fluency	0.10	0.54
	attention/processing speed	-0.22	0.19
	executive function	-0.04	0.83

Note: Values are Spearman's rank correlation coefficient.

Abbreviate: ARMS, at-risk mental state; BACS, Brief Assessment of Cognition in Schizophrenia; CSD, current source density; mGAF, modified Global Assessment Functioning; MTG, middle frontal gyrus; SCoRS, Schizophrenia Cognition Rating Scale.
